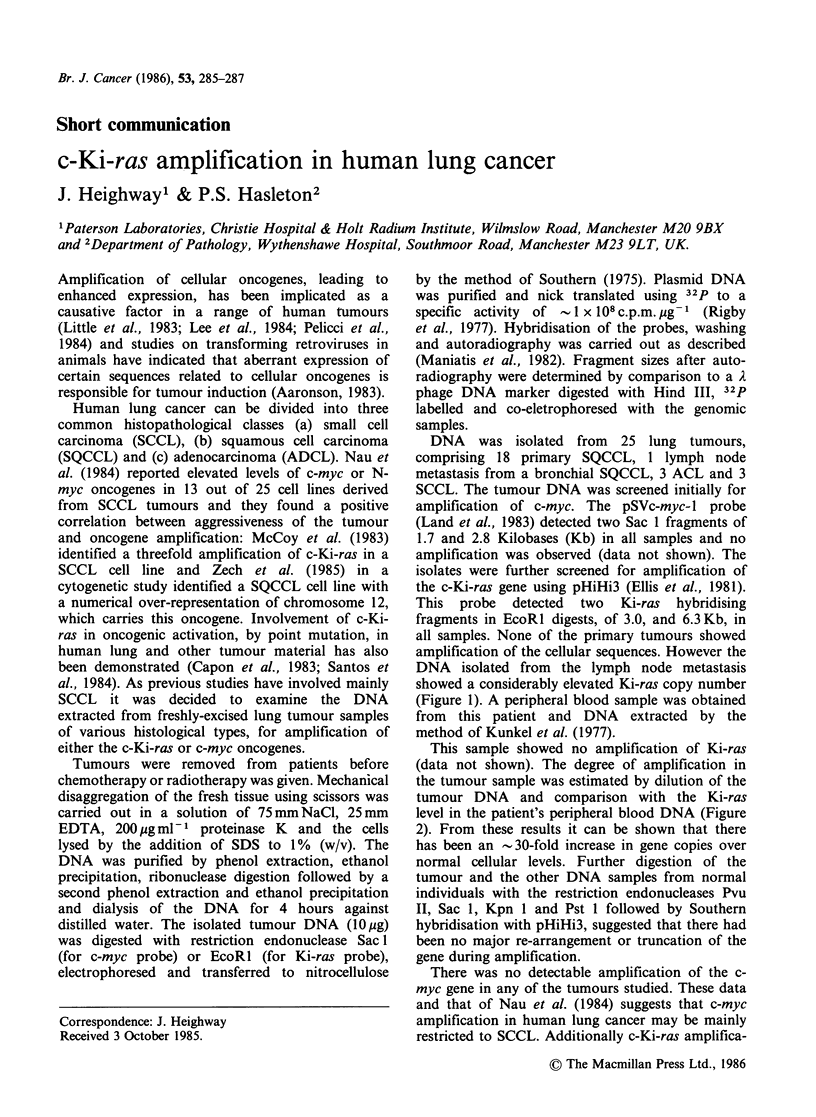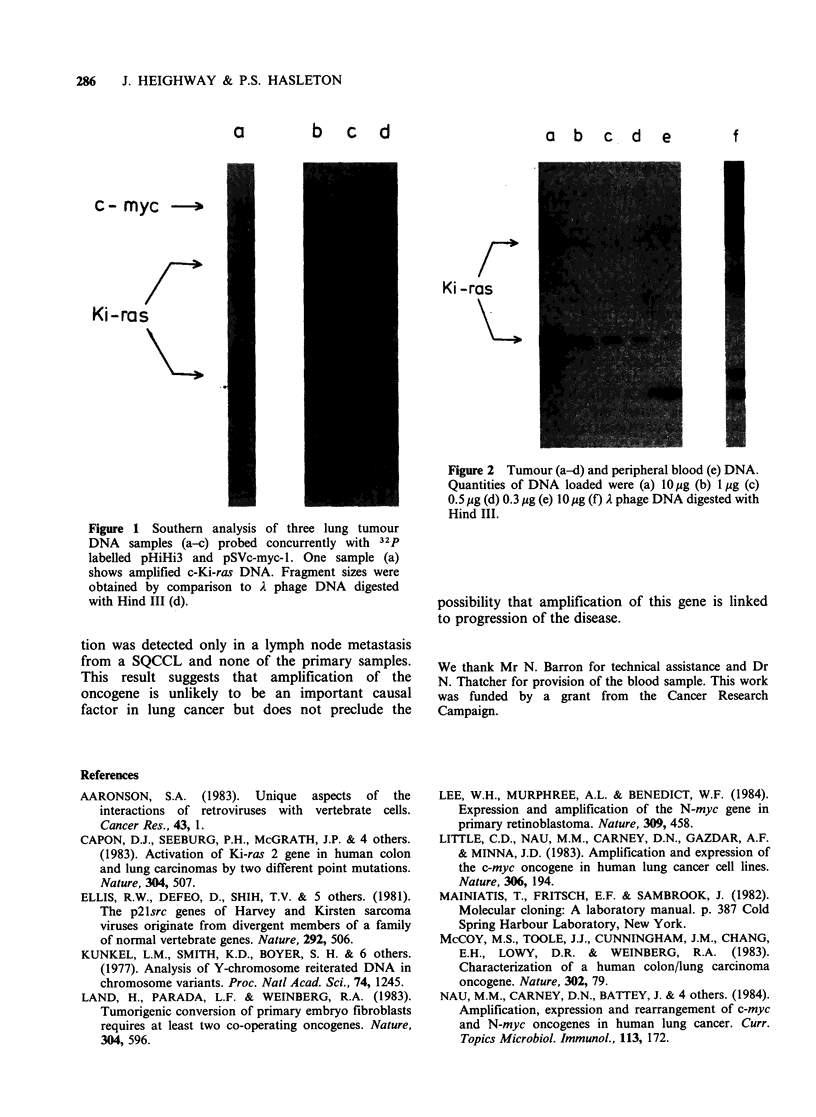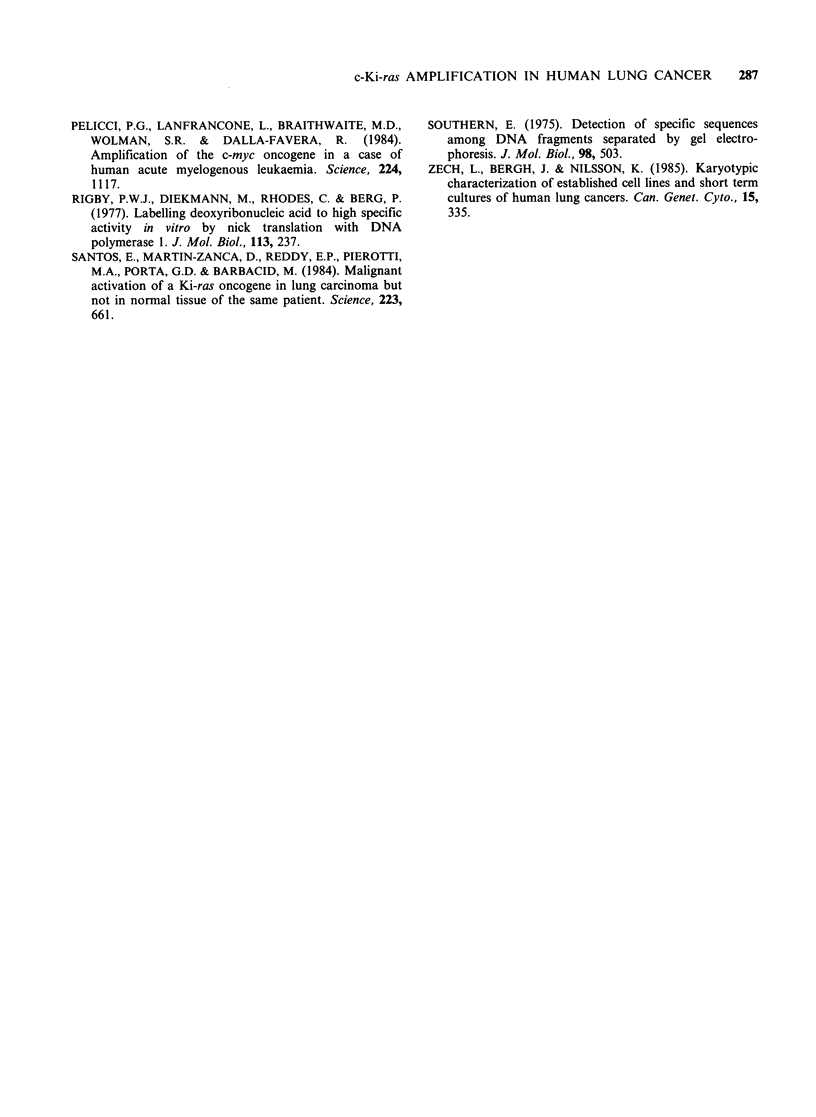# c-Ki-ras amplification in human lung cancer.

**DOI:** 10.1038/bjc.1986.47

**Published:** 1986-02

**Authors:** J. Heighway, P. S. Hasleton

## Abstract

**Images:**


*Br. J. Cancer* (1986), **53**, 285–287

### Short communication

### c-Ki-*ras* amplification in human lung cancer

J. Heighway[1] & P.S. Hasleton[2]

[1]*Paterson Laboratories, Christie Hospital & Holt Radium Institute, Wilmslow Road, Manchester M20 9BX and* [2]*Department of Pathology, Wythenshawe Hospital, Southmoor Road, Manchester M23 9LT, UK.*

Amplification of cellular oncogenes, leading to enhanced expression, has been implicated as a causative factor in a range of human tumours (Little *et al.*, 1983; Lee *et al.*, 1984; Pelicci *et al.*, 1984) and studies on transforming retroviruses in animals have indicated that aberrant expression of certain sequences related to cellular oncogenes is responsible for tumour induction (Aaronson, 1983).

Human lung cancer can be divided into three common histopathological classes (a) small cell carcinoma (SCCL), (b) squamous cell carcinoma (SQCCL) and (c) adenocarcinoma (ADCL). Nau *et al.* (1984) reported elevated levels of c-*myc* or N-*myc* oncogenes in 13 out of 25 cell lines derived from SCCL tumours and they found a positive correlation between aggressiveness of the tumour and oncogene amplification: McCoy *et al.* (1983) identified a threefold amplification of c-Ki-*ras* in a SCCL cell line and Zech *et al.* (1985) in a cytogenetic study identified a SQCCL cell line with a numerical over-representation of chromosome 12, which carries this oncogene. Involvement of c-Ki-*ras* in oncogenic activation, by point mutation, in human lung and other tumour material has also been demonstrated (Capon *et al.*, 1983; Santos *et al.*, 1984). As previous studies have involved mainly SCCL it was decided to examine the DNA extracted from freshly-excised lung tumour samples of various histological types, for amplification of either the c-Ki-*ras* or c-*myc* oncogenes.

Tumours were removed from patients before chemotherapy or radiotherapy was given. Mechanical disaggregation of the fresh tissue using scissors was carried out in a solution of 75 mm NaCl, 25 mm EDTA, $200\,\mu g\,ml^{-1}$ proteinase K and the cells lysed by the addition of SDS to 1% (w/v). The DNA was purified by phenol extraction, ethanol precipitation, ribonuclease digestion followed by a second phenol extraction and ethanol precipitation and dialysis of the DNA for 4 hours against distilled water. The isolated tumour DNA ($10\,\mu g$) was digested with restriction endonuclease Sac 1 (for c-*myc* probe) or EcoR1 (for Ki-*ras* probe), electrophoresed and transferred to nitrocellulose by the method of Southern (1975). Plasmid DNA was purified and nick translated using $^{32}P$ to a specific activity of $\sim 1\times10^{8}$ c.p.m. $\mu g^{-1}$ (Rigby *et al.*, 1977). Hybridisation of the probes, washing and autoradiography was carried out as described (Maniatis *et al.*, 1982). Fragment sizes after autoradiography were determined by comparison to a $\lambda$ phage DNA marker digested with Hind III, $^{32}P$ labelled and co-eletrophoresed with the genomic samples.

DNA was isolated from 25 lung tumours, comprising 18 primary SQCCL, 1 lymph node metastasis from a bronchial SQCCL, 3 ACL and 3 SCCL. The tumour DNA was screened initially for amplification of c-*myc*. The pSVc-*myc*-1 probe (Land *et al.*, 1983) detected two Sac 1 fragments of 1.7 and 2.8 Kilobases (Kb) in all samples and no amplification was observed (data not shown). The isolates were further screened for amplification of the c-Ki-*ras* gene using pHiHi3 (Ellis *et al.*, 1981). This probe detected two Ki-*ras* hybridising fragments in EcoR1 digests, of 3.0, and 6.3 Kb, in all samples. None of the primary tumours showed amplification of the cellular sequences. However the DNA isolated from the lymph node metastasis showed a considerably elevated Ki-*ras* copy number (Figure 1). A peripheral blood sample was obtained from this patient and DNA extracted by the method of Kunkel *et al.* (1977).

This sample showed no amplification of Ki-*ras* (data not shown). The degree of amplification in the tumour sample was estimated by dilution of the tumour DNA and comparison with the Ki-*ras* level in the patient's peripheral blood DNA (Figure 2). From these results it can be shown that there has been an $\sim$30-fold increase in gene copies over normal cellular levels. Further digestion of the tumour and the other DNA samples from normal individuals with the restriction endonucleases Pvu II, Sac 1, Kpn 1 and Pst 1 followed by Southern hybridisation with pHiHi3, suggested that there had been no major re-arrangement or truncation of the gene during amplification.

There was no detectable amplification of the c-*myc* gene in any of the tumours studied. These data and that of Nau *et al.* (1984) suggests that c-*myc* amplification in human lung cancer may be mainly restricted to SCCL. Additionally c-Ki-*ras* amplifica-

Correspondence: J. Heighway
Received 3 October 1985.

© The Macmillan Press Ltd., 1986

**Figure 1** Southern analysis of three lung tumour DNA samples (a–c) probed concurrently with $^{32}P$ labelled pHiHi3 and pSVc-myc-1. One sample (a) shows amplified c-Ki-*ras* DNA. Fragment sizes were obtained by comparison to λ phage DNA digested with Hind III (d).

**Figure 2** Tumour (a–d) and peripheral blood (e) DNA. Quantities of DNA loaded were (a) 10 μg (b) 1 μg (c) 0.5 μg (d) 0.3 μg (e) 10 μg (f) λ phage DNA digested with Hind III.

tion was detected only in a lymph node metastasis from a SQCCL and none of the primary samples. This result suggests that amplification of the oncogene is unlikely to be an important causal factor in lung cancer but does not preclude the possibility that amplification of this gene is linked to progression of the disease.

We thank Mr N. Barron for technical assistance and Dr N. Thatcher for provision of the blood sample. This work was funded by a grant from the Cancer Research Campaign.

### References

AARONSON, S.A. (1983). Unique aspects of the interactions of retroviruses with vertebrate cells. *Cancer Res.*, **43**, 1.

CAPON, D.J., SEEBURG, P.H., McGRATH, J.P. & 4 others. (1983). Activation of Ki-*ras* 2 gene in human colon and lung carcinomas by two different point mutations. *Nature*, **304**, 507.

ELLIS, R.W., DEFEO, D., SHIH, T.V. & 5 others. (1981). The p21*src* genes of Harvey and Kirsten sarcoma viruses originate from divergent members of a family of normal vertebrate genes. *Nature*, **292**, 506.

KUNKEL, L.M., SMITH, K.D., BOYER, S. H. & 6 others. (1977). Analysis of Y-chromosome reiterated DNA in chromosome variants. *Proc. Natl Acad. Sci.*, **74**, 1245.

LAND, H., PARADA, L.F. & WEINBERG, R.A. (1983). Tumorigenic conversion of primary embryo fibroblasts requires at least two co-operating oncogenes. *Nature*, **304**, 596.

LEE, W.H., MURPHREE, A.L. & BENEDICT, W.F. (1984). Expression and amplification of the N-*myc* gene in primary retinoblastoma. *Nature*, **309**, 458.

LITTLE, C.D., NAU, M.M., CARNEY, D.N., GAZDAR, A.F. & MINNA, J.D. (1983). Amplification and expression of the c-*myc* oncogene in human lung cancer cell lines. *Nature*, **306**, 194.

MAINIATIS, T., FRITSCH, E.F. & SAMBROOK, J. (1982). Molecular cloning: A laboratory manual. p. 387 Cold Spring Harbour Laboratory, New York.

McCOY, M.S., TOOLE, J.J., CUNNINGHAM, J.M., CHANG, E.H., LOWY, D.R. & WEINBERG, R.A. (1983). Characterization of a human colon/lung carcinoma oncogene. *Nature*, **302**, 79.

NAU, M.M., CARNEY, D.N., BATTEY, J. & 4 others. (1984). Amplification, expression and rearrangement of c-*myc* and N-*myc* oncogenes in human lung cancer. *Curr. Topics Microbiol. Immunol.*, **113**, 172.

PELICCI, P.G., LANFRANCONE, L., BRAITHWAITE, M.D., WOLMAN, S.R. & DALLA-FAVERA, R. (1984). Amplification of the c-*myc* oncogene in a case of human acute myelogenous leukaemia. *Science*, **224**, 1117.

RIGBY, P.W.J., DIEKMANN, M., RHODES, C. & BERG, P. (1977). Labelling deoxyribonucleic acid to high specific activity *in vitro* by nick translation with DNA polymerase 1. *J. Mol. Biol.*, **113**, 237.

SANTOS, E., MARTIN-ZANCA, D., REDDY, E.P., PIEROTTI, M.A., PORTA, G.D. & BARBACID, M. (1984). Malignant activation of a Ki-*ras* oncogene in lung carcinoma but not in normal tissue of the same patient. *Science*, **223**, 661.

SOUTHERN, E. (1975). Detection of specific sequences among DNA fragments separated by gel electrophoresis. *J. Mol. Biol.*, **98**, 503.

ZECH, L., BERGH, J. & NILSSON, K. (1985). Karyotypic characterization of established cell lines and short term cultures of human lung cancers. *Can. Genet. Cyto.*, **15**, 335.